# Impact of cumulative blood pressure load during early pregnancy on the risk of low birth weight: the BOSHI study

**DOI:** 10.1038/s41440-025-02421-7

**Published:** 2025-10-20

**Authors:** Hiroki Nobayashi, Seiya Izumi, Michihiro Satoh, Noriyuki Iwama, Takahisa Murakami, Go Kanzaki, Yutaro Iwabe, Yuya Suzuki, Mami Ishikuro, Nobuo Tsuboi, Taku Obara, Takayoshi Ohkubo, Yutaka Imai, Takashi Yokoo, Hirohito Metoki

**Affiliations:** 1 Division of Public Health, Hygiene and Epidemiology, Faculty of Medicine, Tohoku Medical and Pharmaceutical University, Sendai, Japan; 2 Division of Nephrology and Hypertension, Department of Internal Medicine, The Jikei University School of Medicine, Tokyo, Japan; 3 Department of Obstetrics and Gynecology, Tohoku University Graduate School of Medicine, Sendai, Japan; 4 Department of Preventive Medicine and Epidemiology, Tohoku Medical Megabank Organization, Tohoku University, Sendai, Japan; 5 Department of Pharmacy, Tohoku Medical and Pharmaceutical University Hospital, Sendai, Japan; 6 Center for Perinatal Medicine, Tohoku University Hospital, Sendai, Japan; 7 Department of Pharmaceutical Sciences, Tohoku University Hospital, Sendai, Japan; 8 Department of Hygiene and Public Health, Teikyo University School of Medicine, Tokyo, Japan; 9 Tohoku Institute for Management of Blood Pressure, Sendai, Japan

**Keywords:** Cumulative blood pressure load, Digital hypertension, Home blood pressure, Implemental hypertension, Morning hypertension

## Abstract

This study investigated the association between cumulative blood pressure (BP) load, defined as the area above a threshold reflecting the duration and magnitude of BP elevation, and low birth weight (LBW). We included 729 pregnant women in this prospective cohort study. Home BP measurements collected at 10 weeks 0 days and 15 weeks 6 days were assessed. The participants were classified into three groups based on their cumulative BP load: (1) no cumulative BP load elevation, (2) isolated cumulative BP load elevation (with normal average BP), and (3) high average BP (average BP above the normal range). Normal ranges (systolic BP < 115 mmHg, diastolic BP < 75 mmHg) were defined following the guidelines; BP load threshold (systolic BP < 104 mmHg, diastolic BP < 62 mmHg) was set using median averages. The mean age at pregnancy was 31.2 years, and 54.4% were primiparas. The mean birth weight was 3060 g; 47 newborns had LBW. The groups with isolated cumulative systolic BP load elevation (risk ratio [RR]: 2.86, 95% confidence interval [CI]: 1.33–6.17) and high average systolic BP (RR: 3.57, 95% CI: 1.38–9.24) showed higher LBW risk than the group without cumulative systolic BP load elevation. Similar associations were observed for cumulative diastolic BP load elevation (RR: 2.22, 95% CI: 1.08–4.58) and high average diastolic BP (RR: 3.35, 95% CI: 1.08–10.34). Our findings highlight the significance of monitoring home BP and the utility of the cumulative BP load in evaluating LBW risk.

**Figure:**
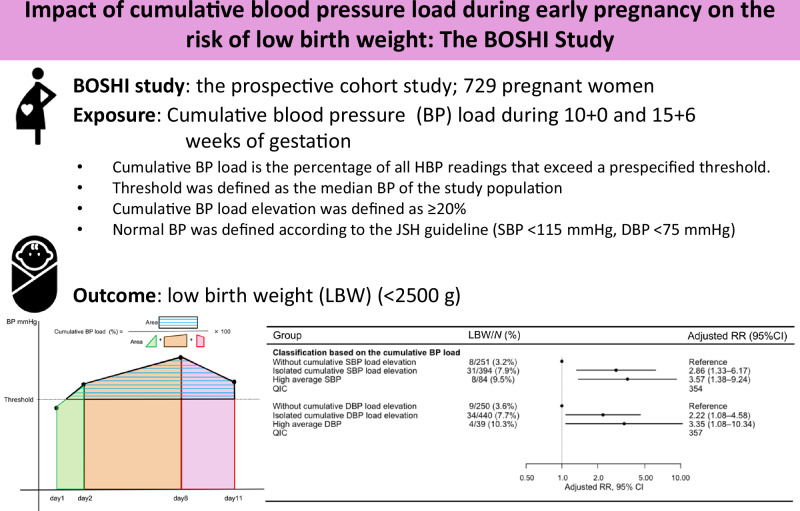
This study evaluated the association between cumulative blood pressure (BP) load elevation during early pregnancy and the risk of low birth weight (LBW). Elevation of both cumulative systolic and diastolic BP load was associated with an increased risk of LBW, even when average BP remained within the normal range

This study evaluated the association between cumulative blood pressure (BP) load elevation during early pregnancy and the risk of low birth weight (LBW). Elevation of both cumulative systolic and diastolic BP load was associated with an increased risk of LBW, even when average BP remained within the normal range

## Abstract (web summary)

This study evaluated the association between cumulative blood pressure (BP) load elevation during early pregnancy and the risk of low birth weight (LBW). Elevation of both cumulative systolic and diastolic BP load was associated with an increased risk of LBW, even when average BP remained within the normal range.

## Introduction

Birth weight is affected by the intrauterine environment, and low birth weight (LBW) is associated with diseases such as hypertension, diabetes, cardiovascular disease, and chronic kidney disease [1–4]. The intrauterine environment includes maternal blood pressure (BP). Hypertensive disorder of pregnancy (HDP), a common perinatal complication, is a risk factor for preterm birth, small-for-gestational age, and LBW [5]. We previously reported that home blood pressure (HBP) before 20 weeks of gestation was associated with infant birth weight in pregnant women without HDP [6].

While proper BP management during pregnancy is crucial, specific criteria for optimal BP levels are lacking, and guidance is generally based on the presence or absence of HDP [7]. Dynamic physiological changes, including hormone level fluctuations, hemodynamics, and vascular resistance, collectively contribute to the J-curve BP trajectory and complicate the determination of optimal BP levels in pregnant women [8–10]. Particularly in early pregnancy, when BP physiologically decreases, the normal BP range defined by the guidelines (systolic BP [SBP] <115 mmHg and diastolic BP [DBP] <75 mmHg) in Japan [11] may not accurately reflect optimal BP levels for pregnant women.

Cumulative BP load has been proposed as an integrated measure of hemodynamic stress exerted on target organs. It is defined as the area above a threshold, incorporating the duration of time during which the BP exceeds the threshold and the magnitude by which it exceeds that level. Per some studies, the cumulative BP load is associated with target organ damage, such as cardiovascular and chronic kidney diseases, in adult populations [12–14]. Although these studies involved calculating the cumulative BP load from the office BP (OBP), the cumulative BP load can also be obtained from the HBP. In obstetrics, several guidelines suggest performing HBP measurements in addition to OBP [15, 16]. Moreover, our previous study revealed that the HBP is superior to the OBP in predicting infant birth weight [6]. Hence, we hypothesized that the cumulative BP load calculated from HBP readings may be a valuable indicator for assessing LBW risk as it accounts for dynamic changes in BP during pregnancy. Therefore, this study aimed to investigate whether elevated cumulative BP load during early pregnancy (between 10 + 0 and 15 + 6 weeks of gestation, the guideline-recommended screening period [15, 16]) is associated with an increased LBW risk.

Point of view

- Clinical relevance
  An elevated cumulative blood pressure load during early pregnancy, even within the normotensive range, is associated with an increased risk of low birth weight.
- Future direction
  The evaluation of the relationship between elevated cumulative blood pressure load and placental growth factor is warranted.
- Consideration for the Asian population
  Evaluating cumulative blood pressure load may be particularly useful in Asian pregnant women, who tend to have lower blood pressure during early pregnancy and a higher frequency of low birth weight compared with Western women.

## Methods

### Study design and population

**Fig. 1:**
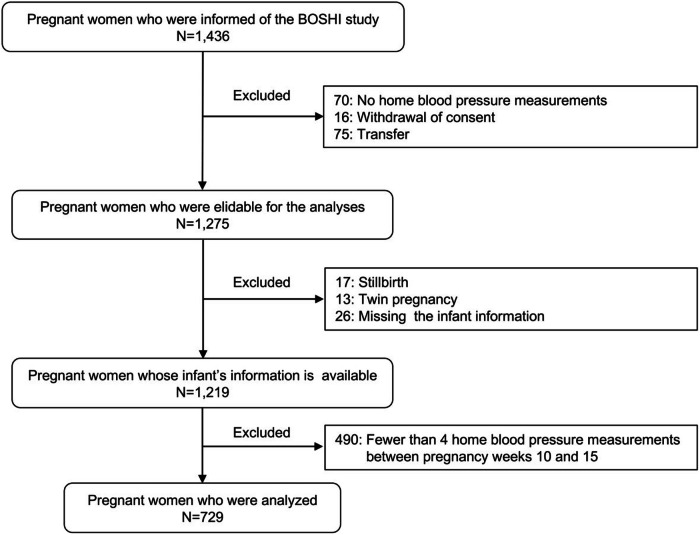
Flow chart of the study

This study was part of the Babies and their Parents’ Longitudinal Observation in Suzuki Memorial Hospital on the Intrauterine Period (BOSHI) study conducted at Suzuki Memorial Hospital, an obstetric and gynecologic hospital in Sendai City, Miyagi Prefecture, Japan. The study procedures and protocols were approved by the Institutional Review Board of Tohoku Medical and Pharmaceutical University, Tohoku University School of Medicine and the Hospital Review Board of Suzuki Memorial Hospital. The details of the BOSHI study have been described previously [6, 17, 18]. Written informed consent was obtained from the participants before their inclusion in the study. Between October 16, 2006, and October 7, 2011, 1,436 pregnant women were informed of the BOSHI study. After excluding pregnant women who did not undergo HBP measurement, withdrew consent, and were transferred, 1275 pregnant women were eligible for the analyses. Among them, 1219 pregnant women with singleton pregnancies whose infant data were available were included. Finally, 729 pregnant women were analyzed after excluding those who underwent HBP measurement fewer than four times between 10 weeks 0 days and 15 weeks 6 days (Fig. 1).

### Clinical data

Data on maternal age, height, pre-pregnancy body weight (BW), parity, pre-pregnancy smoking status, and alcohol consumption were collected using questionnaires and surveys by midwives. The pre-pregnancy body mass index (BMI) was calculated as the pre-pregnancy BW in kilograms divided by the square of the maternal height in meters. HDP history or complications and information on assisted reproductive technology were obtained from medical records [6, 17, 18]. HDP was diagnosed following the guidelines of the Japanese Society for the Study of Hypertension in Pregnancy [19]. Infant information such as birth weight, delivery week, and sex was collected from medical records.

### HBP measurement and cumulative BP load

Health providers, including physicians, midwives, and pharmacists, trained participants on HBP measurement techniques after enrollment. HBP was measured following the Japanese Society of Hypertension (JSH) guidelines for HBP self-monitoring [11]. Participants were asked to measure their HBP in their upper arms every morning, within 1 h of waking up, after micturition, before breakfast, while seated, and after resting for at least 1 min. HBP was measured using an HEM-747IC or HEM-7080UC (Omron Healthcare, Kyoto, Japan), a semiautomatic device based on the cuff-oscillometric method, and digital readings of SBP and DBP pulse rates were generated. The first BP measurement recorded each day during the observation period was used for analysis. The averages of the measured home SBPs and DBPs were calculated for analysis. Because the number of HBP measurements before 10 weeks of gestation was insufficient for analysis, these data were excluded.

Daily SBP and DBP values were plotted and connected using straight lines, and the total area under the straight-line segments was calculated. Threshold values were set separately for SBP and DBP, and the areas above these thresholds were computed. The threshold for BP load was set at the median average BP among pregnant women, excluding those with chronic hypertension. In the absence of an established cutoff value, a cumulative BP load exceeding 20%, which represented the first quartile excluding 0% and 100%, was defined as elevated. The cumulative BP load was defined as the percentage of the area above the threshold relative to the total area under the straight-line segments [12–14]. The details of the calculation of the cumulative BP load are shown in Supplementary Fig. 1.

### Outcome

The primary outcome of this study was LBW, defined as an infant birth weight <2500 g. LBW is a comprehensive indicator that reflects shortened gestational age and impaired fetal growth. Because the cumulative BP load in early pregnancy can affect placental function and fetal development through multiple pathways, LBW provides a clinically relevant and integrative measure of these effects.

### Statistical analysis

Continuous variables are presented as mean ± standard deviation (SD) or median with interquartile range (IQR), as appropriate. Categorical variables are described using frequency and proportion. The correlation between cumulative BP load and average BP was assessed using Spearman’s rank correlation coefficient. Based on the cumulative BP load and average BP, pregnant women were categorized into three groups: (1) without cumulative BP load elevation, (2) isolated cumulative BP load elevation (with normal average BP), and (3) high average BP (average BP equal to or above the high-normal range [SBP ≥ 115 mmHg and/or DBP ≥ 75 mmHg]). The risk ratios for LBW were estimated using Poisson regression with robust standard errors, with the group without cumulative BP load elevation as the reference. Risk estimation was adjusted for covariates, including maternal age at gestation, pre-pregnancy BMI, pre-pregnancy smoking status, primiparity, and HDP history.

We conducted several sensitivity analyses. Furthermore, we assessed the association between average BP and LBW risk using multiple models and compared the Quasi-likelihood under the Independence model Criterion (QIC) between cumulative BP load and average BP models. The average BP risk models included four components: sample size distribution matching that of the primary analysis, average BP tertiles, JSH guidelines, and median average BP. In the first two analyses, participants were classified into three groups according to the ascending average BP. In the first component of the average BP risk model, the classification was performed to match the sample size distribution with that of the primary analysis. The classification in the second component of the model was based on the cutoffs of the average BP tertiles. To examine the nonlinear association with LBW risk, we performed Poisson regression with robust standard errors by incorporating restricted cubic splines. For cumulative BP load, the risk at the minimum value was used as the reference, whereas for average BP, the median value served as the reference. In addition, cumulative BP load elevation was alternatively defined using 10% and 30% cutoff values. We also performed two additional analyses: one restricted to women without chronic hypertension, and another restricted to those with ≥8 HBP measurements (i.e., excluding the lowest quartile of measurement frequency). These risk estimates were compared with those of the primary model using QIC.

Missing values were imputed using multiple imputations performed over 10 iterations. Multiple imputation and Poisson regression analyses were performed using R software (version 4.3.3; R Foundation for Statistical Computing, Vienna, Austria), and other analyses were conducted using SAS software (version 9.4 1M7; SAS Institute, Cary, NC, USA).

## Results

### Maternal and neonatal characteristics

Table 1 presents the maternal and neonatal characteristics. The mean age at pregnancy was 31.2 years, and 54.4% were primiparas. Additionally, 89 pregnant women developed HDP during pregnancy, 13 of whom had chronic hypertension. The mean delivery weeks was 39.6 weeks, and 3.2% of the deliveries were preterm. Furthermore, the mean birth weight was 3060 g, and 47 newborns had LBW. The median cumulative SBP and DBP loads were 49% and 51%, respectively. Similarly, the median average SBP and DBP were 103.9 and 61.8 mmHg, respectively. The median number of HBP measurements was 12. A summary of the characteristics based on the cumulative BP load is presented in Supplementary Tables 1 and 2.

**Table 1:** Maternal and neonatal baseline characteristics

Variables	Values (*N* = 729)
Maternal characteristics	
Age at the time of consent, years, mean ± SD	31.2 ± 4.7
Height, cm, mean ± SD	158.4 ± 5.1
Pre-pregnancy body weight, kg, mean ± SD	54.4 ± 9.0
Pre-pregnancy BMI, kg/m2, mean ± SD	21.7 ± 3.4
Primipara, *n* (%)	422 (57.9)
Smoking, *n* (%)	
No smoking before conception	619 (85.5)
Until conception was recognized	82 (11.2)
Smoking during pregnancy	28 (3.9)
Alcohol intake, *n* (%)	
No alcohol intake before conception	443 (60.8)
Until conception was recognized	278 (38.1)
Alcohol intake during pregnancy	8 (1.1)
HDP in prior pregnancy, *n* (%)	16 (2.2)
HDP during pregnancy, *n* (%)	89 (12.2)
Chronic Hypertension, *n* (%)	13 (1.8)
Assisted reproductive technology, *n* (%)	2 (0.3)
Delivery weeks, weeks, mean ± SD	39.6 ± 1.4
Preterm delivery, *n* (%)	23 (3.2)
Neonatal characteristics	
Male Sex, *n* (%)	351 (48.2)
Birth weight, g ± SD	3060 ± 392
Low birth weight (<2500 g), *n* (%)	47 (6.5)
Home BP monitoring	
Cumulative SBP load, %, median [IQR]	49 [7–96]
Cumulative DBP load, %, median [IQR]	51 [8–96]
Average SBP, mmHg, median [IQR]	103.9 [98.5–109.9]
Average DBP, mmHg, median [IQR]	61.8 [57.6–66.9]
Number of BP measurements, median [IQR]	12 [8–19]

*BMI* body mass index, *BP* blood pressure, *DBP* diastolic blood pressure, *HDP* hypertensive disorders of pregnancy, *IQR* interquartile range, *SBP* systolic blood pressure, *SD* standard deviation

### Cumulative BP load and LBW risk

**Fig. 2:**
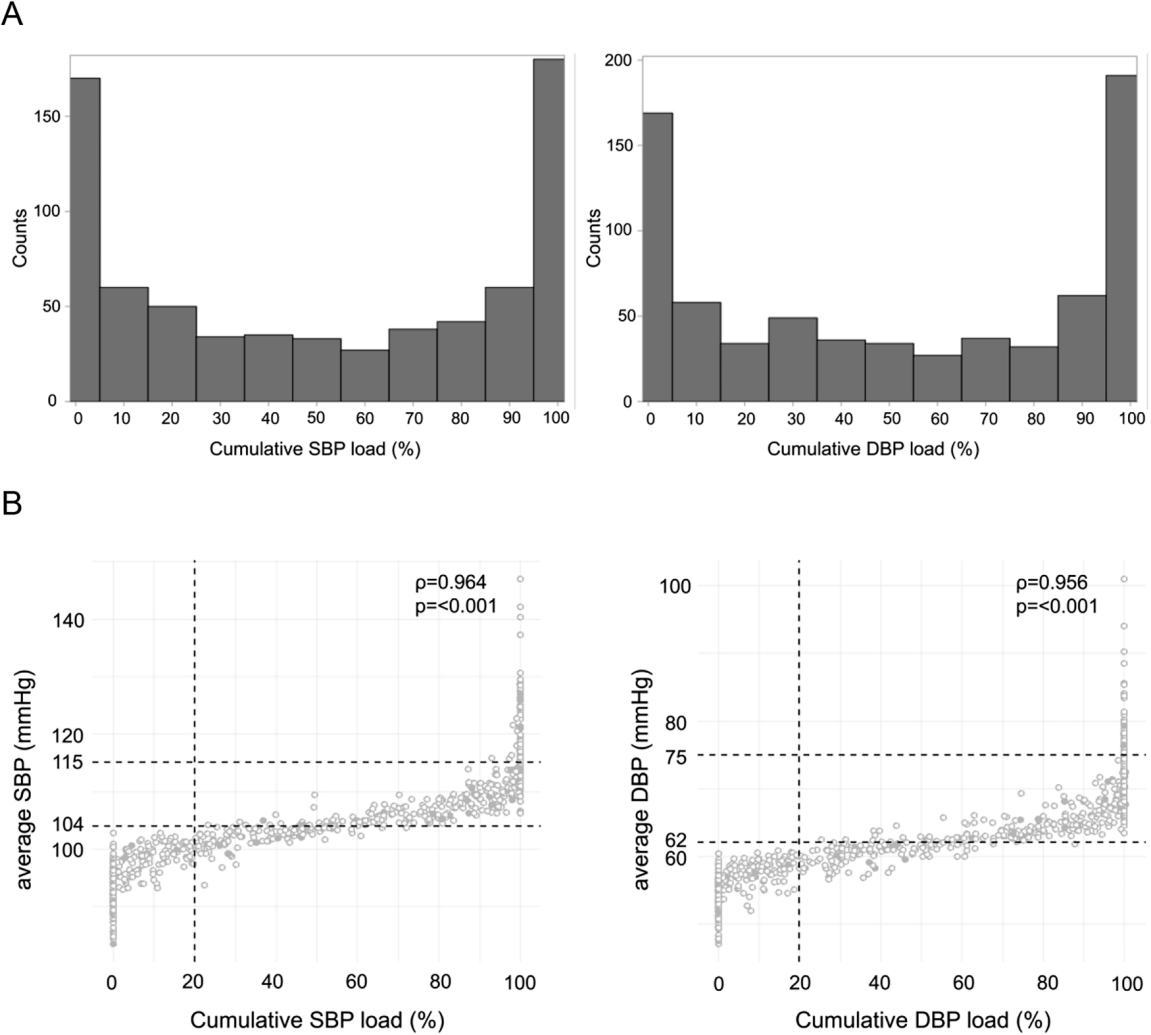
The distribution of cumulative blood pressure load and its association with average blood pressure. **A** Histograms of the cumulative systolic and diastolic blood pressure load. **B** Scatter plots illustrating the association between the cumulative blood pressure load and average blood pressure. Each point represents a pregnant woman; filled circles indicate those who delivered a low birth weight. average SBP = 104 mmHg and average DBP = 62 mmHg represent the median values of average systolic and diastolic blood pressure, respectively. average SBP = 115 mmHg and average DBP = 75 mmHg correspond to the upper limits of the normal blood pressure according to the guideline of Japanese Society of Hypertension

For cumulative SBP and DBP loads, most pregnant women were distributed at 0% and 100% (Fig. 2A). Among those with a cumulative SBP load <90%, no individual had an average SBP exceeding the normal threshold of 115 mmHg. Similarly, for cumulative DBP load, no woman with a cumulative DBP load <100% had an average DBP exceeding the normal threshold of 75 mmHg (Fig. 2B). Cumulative BP load significantly correlated with average BP for SBP (*ρ* = 0.964) and DBP (*ρ* = 0.956).

**Fig. 3:**
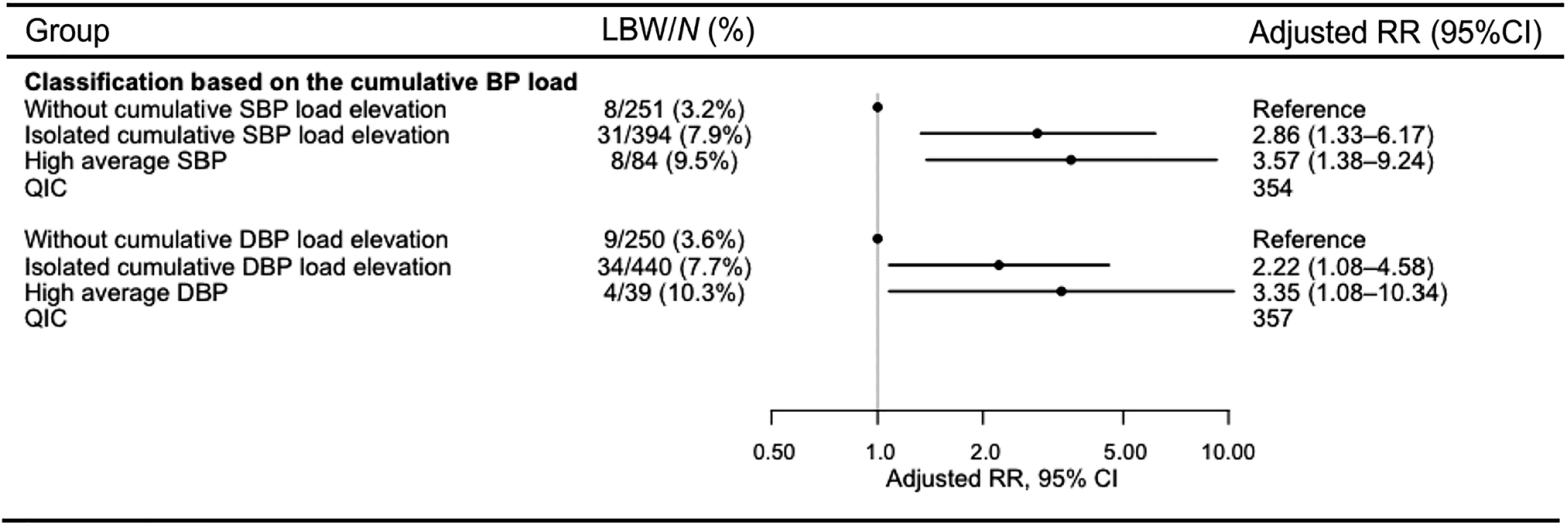
Risk assessment of low birth weight based on the cumulative blood pressure load. Risk ratios were adjusted for maternal age at gestation, pre-pregnancy body mass index, pre-pregnancy smoking status, primiparity, and history of hypertensive disorders of pregnancy. Abbreviations: BP blood pressure, CI confidence interval, DBP diastolic blood pressure, LBW low birth weight, QIC Quasi-likelihood under the Independence model Criterion, RR risk ratio, SBP systolic blood pressure

The groups with isolated SBP load elevation (risk ratio [RR]: 2.86, 95% confidence interval [CI]: 1.33–6.17) and high average SBP (RR: 3.57, 95% CI: 1.38–9.24) showed higher LBW risk than the group without cumulative SBP load elevation. Similarly, elevated LBW risks were observed in isolated cumulative DBP (RR: 2.22, 95% CI: 1.08–4.58) and high average DBP (RR: 3.35, 95% CI: 1.08–10.34) groups (Fig. 3).

### Sensitivity analyses

**Fig. 4:**
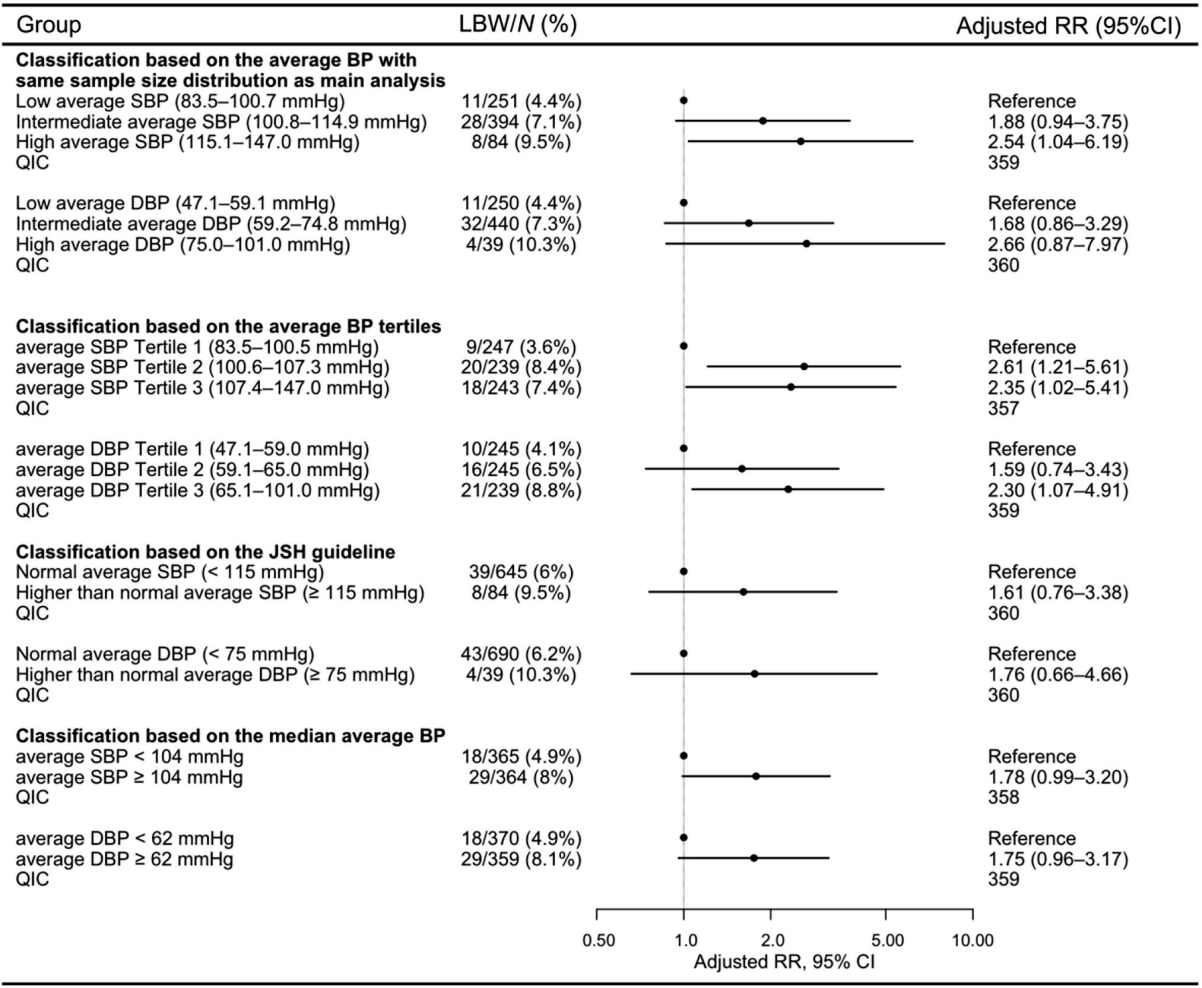
Risk assessment of low birth weight based on the average blood pressure. Risk ratios were adjusted for maternal age at gestation, pre-pregnancy body mass index, pre-pregnancy smoking status, primiparity, and history of hypertensive disorders of pregnancy. Abbreviations: BP blood pressure, CI confidence interval, DBP diastolic blood pressure, JSH Japanese Society of Hypertension, LBW low birth weight, QIC Quasi-likelihood under the Independence model Criterion, RR risk ratio, SBP systolic blood pressure

When pregnant women were divided into three (low, intermediate, and high) groups based on average BP values (with a sample size distribution equal to that in the primary analysis), using the low group as the reference, a significant increase in LBW risk was observed only in the high average SBP group (RR: 2.54, 95% CI: 1.04–6.19). In analyses based on average BP tertiles (T1–T3), with T1 as the reference, average SBP significantly increased LBW risk in T2 (RR: 2.61, 95% CI: 1.21–5.61) and T3 (RR: 2.35, 95% CI: 1.02–5.41), with a higher risk in T2. For average DBP, only T3 was associated with a significantly increased risk of LBW (RR: 2.30, 95% CI: 1.07–4.91). Compared with normal BP, based on the JSH guidelines, high-normal BP and higher categories showed no significant increase in LBW risk for average SBP or DBP. Similarly, no significant increase in risk was observed when comparing women above and below the median average BP in this cohort (Fig. 4). Risk analyses based on the average BP classification showed higher QIC values than those based on the cumulative BP load (Figs. 3 and 4). Continuous analysis revealed a nonlinear association between cumulative SBP load and LBW risk, with the risk increasing and peaking at ~40%. The cumulative DBP load showed a gradual increase of ~40% in LBW risk, followed by a plateau (Supplementary Fig. 2A). In contrast, the average SBP demonstrated a gradual increase in LBW risk, whereas the average DBP showed a nonlinear association, peaking at ~70–75 mmHg (Supplementary Fig. 2B).

A significant increase in LBW risk persisted in the cumulative BP load elevation group even when the cutoff was adjusted to 10% or 30%. Based on the QIC comparisons, the lowest QIC was observed at 20% (primary analysis) and 30% for the cumulative SBP and DBP loads, respectively (Fig. 3 and Supplementary Fig. 3). Similar trends were observed when restricting the analysis to pregnant women without chronic hypertension or those with ≥8 BP measurements. The QICs decreased markedly among pregnant women who had eight or more BP measurements (Supplementary Fig. 3).

## Discussion

Elevated cumulative BP load during early pregnancy was associated with an increased risk of LBW, even when the average BP remained within the normal range. In addition, cumulative SBP and DBP loads demonstrated a nonlinear association with LBW risk, which became significant when the cumulative BP load exceeded ~20%. These outcomes reveal that even a slight elevation in the cumulative BP load may contribute to an increased LBW risk.

Notably, the BP load threshold was determined based on the median average BP of the participants. Cumulative SBP and DBP loads were associated with a significantly increased risk of LBW, starting at ~20%, with the risk curve peaking at ~40%. However, based on the association between cumulative BP load and average BP, most pregnant women with a cumulative BP load <40% had average BP levels below the median of the study population. Furthermore, average BP readings above the normal range and cohort median were not associated with increased LBW risk, indicating that the reference group included high-risk pregnant women. Using the median value as the reference, continuous analysis of LBW risk based on average BP showed no significant differences across the full range for average SBP or DBP, supporting this interpretation. This suggests that managing the BP below the guideline-defined normal range may be insufficient during early pregnancy.

Additionally, we performed similar analyses by categorizing the average BP into three groups, including a model with a sample size distribution matching that of the primary analysis and another based on average BP tertiles. An increased risk of LBW was observed in the highest BP group compared with the lowest BP group. Given the strong correlation between average BP and cumulative BP load, this finding was expected. However, all models based on average BP had higher QIC values than those from the primary analysis involving the cumulative BP load classification. Notably, for DBP, although the sample size distribution was the same as that in the primary analysis, the high average DBP group was not significantly associated with LBW risk when classified using average DBP, whereas it was significantly associated with LBW risk when classified using cumulative DBP load. These findings suggest that evaluating early pregnancy BP using cumulative BP load rather than average BP may allow for a more accurate assessment of LBW risk. In clinical practice, this underscores the significance of monitoring daily HBP measurements and carefully assessing days exceeding the threshold. This observation is also consistent with that of a previous study, indicating the superiority of HBP monitoring over OBP measurements [6]. The analysis restricted to participants with ≥8 HBP measurements showed the lowest QIC, indicating the significance of obtaining multiple HBP measurements during early pregnancy.

The mechanisms by which cumulative BP load is associated with increased LBW risk remain unclear. A possible physiological pathway involves abnormal increases in soluble fms-like tyrosine kinase-1 (sFlt-1) and soluble endoglin (sEng), accompanied by a decrease in placental growth factor (PlGF), which have been implicated in the pathogenesis of preeclampsia [20–22]. Per a previous study, these circulating angiogenic factors correlated with the mean BP [23], suggesting that the cumulative BP load may contribute to placental insufficiency through alterations in angiogenic factors.

This study has some limitations. First, the study was conducted at a single hospital. Although the external validity possibly supports our results [24], the study findings need to be validated in other populations. Second, this study included only pregnant women who measured their HBP at least four times, which may have introduced a selection bias. Women who did not measure their HBP may have had higher BP levels than those in the study population, suggesting that such individuals should also be considered when determining appropriate BP load thresholds. Third, we did not measure angiogenic factors, such as sFlt-1, sEng, and PlGF. Therefore, the mechanisms by which cumulative BP load elevation causes LBW remain unclear. Further investigations are needed to clarify the association between cumulative BP load elevation and LBW. Fourth, this study lacked information regarding the use of medications, including antihypertensive drugs, during early pregnancy. However, the cumulative BP load reflects the overall effect of these factors on BP during pregnancy, and remains useful for assessing the risk of LBW in pregnant women receiving those drugs.

### Perspective of Asia

Racial differences in BP during pregnancy have been reported, with Asian women exhibiting lower BP in early pregnancy compared to their Western counterparts [25]. Therefore, it remains to be clarified whether cumulative BP load is also helpful in predicting the risk of LBW among Western populations. On the other hand, a study has reported that Asians have a higher frequency of LBW compared with Western populations [26], suggesting that the evaluation of cumulative BP load may be particularly valuable in Asian populations.

An elevated cumulative BP load during early pregnancy, even within the normotensive range, was associated with an increased risk of LBW. Our findings highlight the significance of monitoring HBP and the utility of the cumulative BP load in evaluating LBW risk.

## Supplementary information

Supplementary information
