# Supplementary material for: Impact of cumulative blood pressure load during early pregnancy on the risk of low birth weight: the BOSHI study
Source: Hypertens Res. 2025 Oct 20;49(2):550–9. doi: 10.1038/s41440-025-02421-7 (PMC12823421; doi:10.1038/s41440-025-02421-7)
Supplement: Supplementary file 1 — Supplementary information [file 41440_2025_2421_MOESM1_ESM.docx]

Supplementary Table 1. Characteristics according to the cumulative systolic blood pressure load

| Variables | Without cumulative SBP load elevation | Isolated cumulative SBP load elevation | High average SBP |
| --- | --- | --- | --- |
| N | 251 | 394 | 84 |
| Maternal characteristics |  |  |  |
| Age at the time of consent, years, mean ± SD | 31.4 ± 4.2 | 30.8 ± 4.8 | 32.0 ± 5.7 |
| Height, cm, mean ± SD | 158.3 ± 5.1 | 158.3 ± 5.1 | 158.9 ± 5.1 |
| Pre-pregnancy body weight, kg, mean ± SD | 51.1 ± 6.2 | 54.8 ± 8.5 | 62.7 ± 12.1 |
| Pre-pregnancy BMI, kg/m^2,^ mean ± SD | 20.4 ± 2.2 | 21.9 ± 3.3 | 24.8 ± 4.8 |
| Primipara, n (%) | 153 (61.0) | 222 (56.4) | 47 (56.0) |
| Smoking, n (%) |  |  |  |
| No smoking before conception | 226 (90.4) | 320 (82.1) | 73 (86.9) |
| Until conception was recognized | 19 (7.6) | 52 (13.3) | 6 (7.1) |
| Smoking during pregnancy | 5 (2.0) | 18 (4.6) | 5 (6.0) |
| Alcohol intake, n (%) |  |  |  |
| No alcohol intake before conception | 139 (55.4) | 241 (61.2) | 63 (75.0) |
| Until conception was recognized | 110 (43.8) | 147 (37.3) | 21 (25.0) |
| Alcohol intake during pregnancy | 2 (0.8) | 6 (1.5) | 0 (0) |
| HDP in prior pregnancy, n (%) | 1 (0.4) | 8 (2.0) | 7 (8.3) |
| HDP during pregnancy, n (%) | 10 (4.0) | 43 (10.9) | 36 (42.9) |
| Chronic Hypertension, n (%) | 0 (0) | 3 (0.8) | 10 (11.9) |
| Assisted reproductive technology, n (%) | 0 (0) | 2 (0.5) | 0 (0) |
| Delivery weeks, weeks, mean ± SD | 39.8 ± 1.1 | 39.6 ± 1.6 | 39.5 ± 1.5 |
| Preterm delivery, n (%) | 3 (1.2) | 12 (3.1) | 8 (9.5) |
| Neonatal characteristics |  |  |  |
| Male Sex, n (%) | 112 (44.6) | 194 (49.2) | 45 (53.6) |
| Birth weight, g ± SD | 3105 ± 357 | 3043 ± 404 | 3043 ± 427 |
| Low birth weight (< 2500 g), n (%) | 8 (3.2) | 31 (7.9) | 8 (9.5) |
| Home BP monitoring |  |  |  |
| Cumulative SBP load, %, median [IQR] | 0 [0–8] | 75 [45–94] | 100 [100–100] |
| Cumulative DBP load, %, median [IQR] | 6 [0–29] | 74 [33–99] | 100 [98–100] |
| Average SBP, mmHg, median [IQR] | 96.2 [93.0–98.6] | 106.1 [103.5–109.9] | 121.4 [116.7–124.8] |
| Average DBP, mmHg, median [IQR] | 57.4 [54.3–60.3] | 63.7 [60.1–67.3] | 73.5 [69.1–77.9] |
| Number of BP measurements, median [IQR] | 12 [8–20] | 12 [7–19] | 11 [8–18] |

Abbreviations: BMI, body mass index; BP, blood pressure; DBP, diastolic blood pressure; HDP, hypertensive disorders of pregnancy; IQR, interquartile range; SBP, systolic blood pressure; SD, standard deviation

Supplementary Table 2. Characteristics according to the cumulative diastolic blood pressure load

| Variables | Without cumulative DBP load elevation | Isolated cumulative DBP load elevation | High average DBP |
| --- | --- | --- | --- |
| N | 250 | 440 | 39 |
| Maternal characteristics |  |  |  |
| Age at the time of consent, years, mean ± SD | 30.9 ± 4.7 | 31.3 ± 4.7 | 32.0 ± 5.0 |
| Height, cm, mean ± SD | 158.4 ± 4.9 | 158.4 ± 5.1 | 157.6 ± 5.3 |
| Pre-pregnancy body weight, kg, mean ± SD | 51.9 ± 6.2 | 54.9 ± 9.0 | 65.4 ± 14.2 |
| Pre-pregnancy BMI, kg/m^2,^ mean ± SD | 20.7 ± 2.2 | 21.9 ± 3.5 | 26.3 ± 11.2 |
| Primipara, n (%) | 147 (58.8) | 254 (57.3) | 21 (53.9) |
| Smoking, n (%) |  |  |  |
| No smoking before conception | 217 (86.8) | 371 (85.1) | 31 (81.6) |
| Until conception was recognized | 27 (10.8) | 47 (10.8) | 4 (10.5) |
| Smoking during pregnancy | 7 (2.8) | 18 (4.1) | 3 (7.9) |
| Alcohol intake, n (%) |  |  |  |
| No alcohol intake before conception | 140 (56.0) | 271 (61.6) | 32 (82.1) |
| Until conception was recognized | 109 (43.6) | 162 (36.8) | 7 (18.0) |
| Alcohol intake during pregnancy | 1 (0.4) | 7 (1.6) | 0 (0) |
| HDP in prior pregnancy, n (%) | 1 (0.4) | 11 (2.5) | 4 (10.3) |
| HDP during pregnancy, n (%) | 4 (1.6) | 62 (14.1) | 23 (59.0) |
| Chronic Hypertension, n (%) | 0 (0) | 5 (1.2) | 8 (21.1) |
| Assisted reproductive technology, n (%) | 0 (0) | 2 (0.5) | 0 (0) |
| Delivery weeks, weeks, mean ± SD | 39.7 ± 1.5 | 39.6 ± 1.4 | 39.3 ± 1.6 |
| Preterm delivery, n (%) | 2 (0.8) | 15 (3.4) | 6 (15.4) |
| Neonatal characteristics |  |  |  |
| Male Sex, n (%) | 122 (48.8) | 211 (48.0) | 18 (46.2) |
| Birth weight, g ± SD | 3102 ± 380 | 3035 ± 389 | 3067 ± 481 |
| Low birth weight (< 2500 g), n (%) | 9 (3.6) | 34 (7.7) | 4 (10.3) |
| Home BP monitoring |  |  |  |
| Cumulative SBP load, %, median [IQR] | 3 [0–29] | 75 [29–98] | 100 [100–100] |
| Cumulative DBP load, %, median [IQR] | 4 [0–8] | 83 [51–100] | 100 [100–100] |
| Average SBP, mmHg, median [IQR] | 97.6 [93.5–101.9] | 106.7 [102.0–111.1] | 123.2 [116.6–128.0] |
| Average DBP, mmHg, median [IQR] | 55.8 [54.0–58.0] | 65.0 [61.8–67.8] | 80.4 [76.7–83.3] |
| Number of BP measurements, median [IQR] | 12 [7–17] | 12 [8–20] | 13 [8–22] |

Abbreviations: BMI, body mass index; BP, blood pressure; DBP, diastolic blood pressure; HDP, hypertensive disorders of pregnancy; IQR, interquartile range; SBP, systolic blood pressure; SD, standard deviation


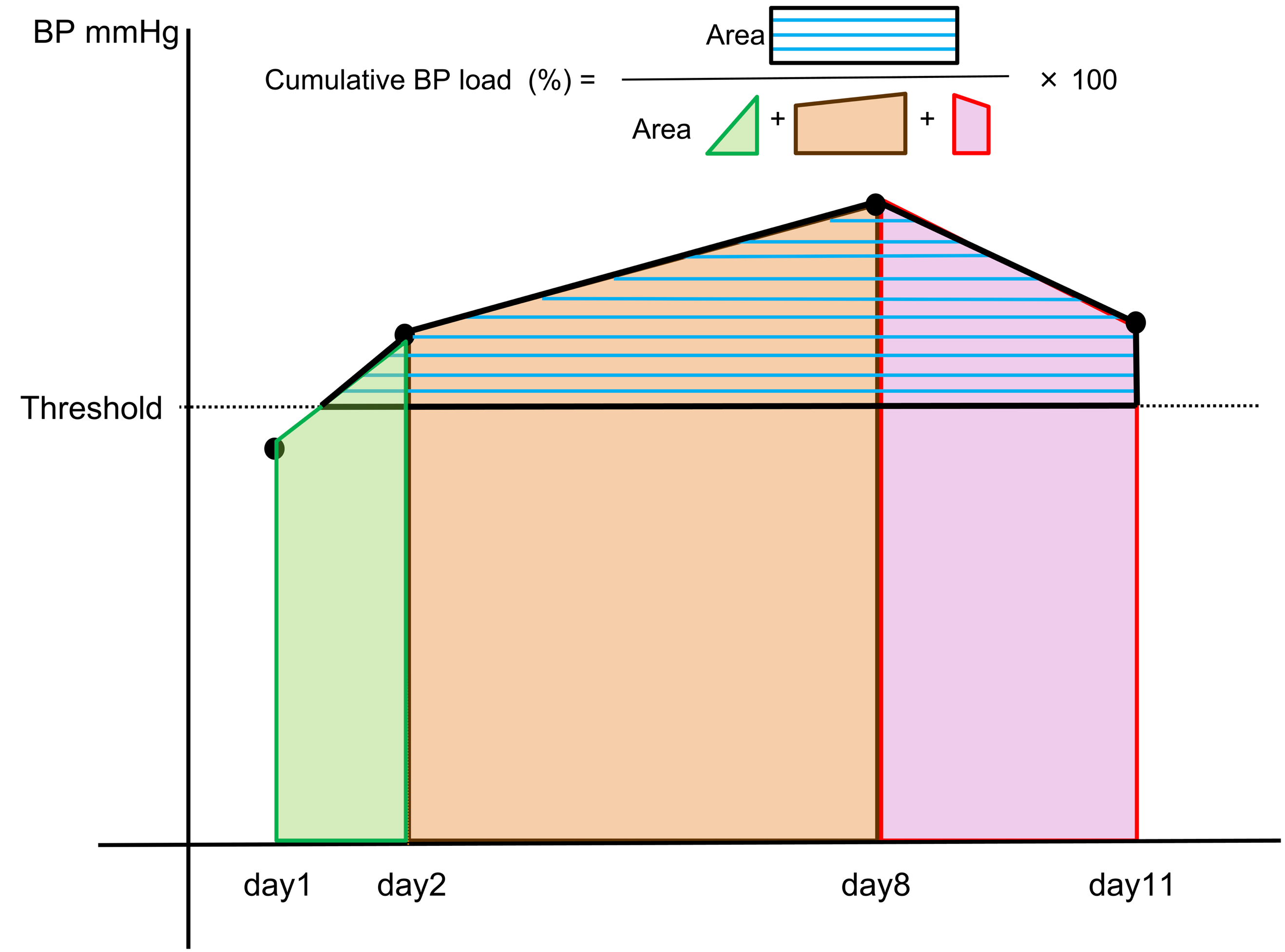


Supplementary FIgure 1. Calculation of cumulative blood pressure load. This figure illustrates an example of a pregnancy woman who measured home blood pressure on days 1, 2, 8, and 11. Each blood pressure measurement is connected by straight lines to form trapezoids between consecutive time points (shown in green, orange, and pink). The blood pressure load is defined as the area exceeding the predefined threshold (horizontal blue line).
The cumulative blood pressure load (%) is calculated as: (Area above the threshold / Total trapezoid area) × 100

A


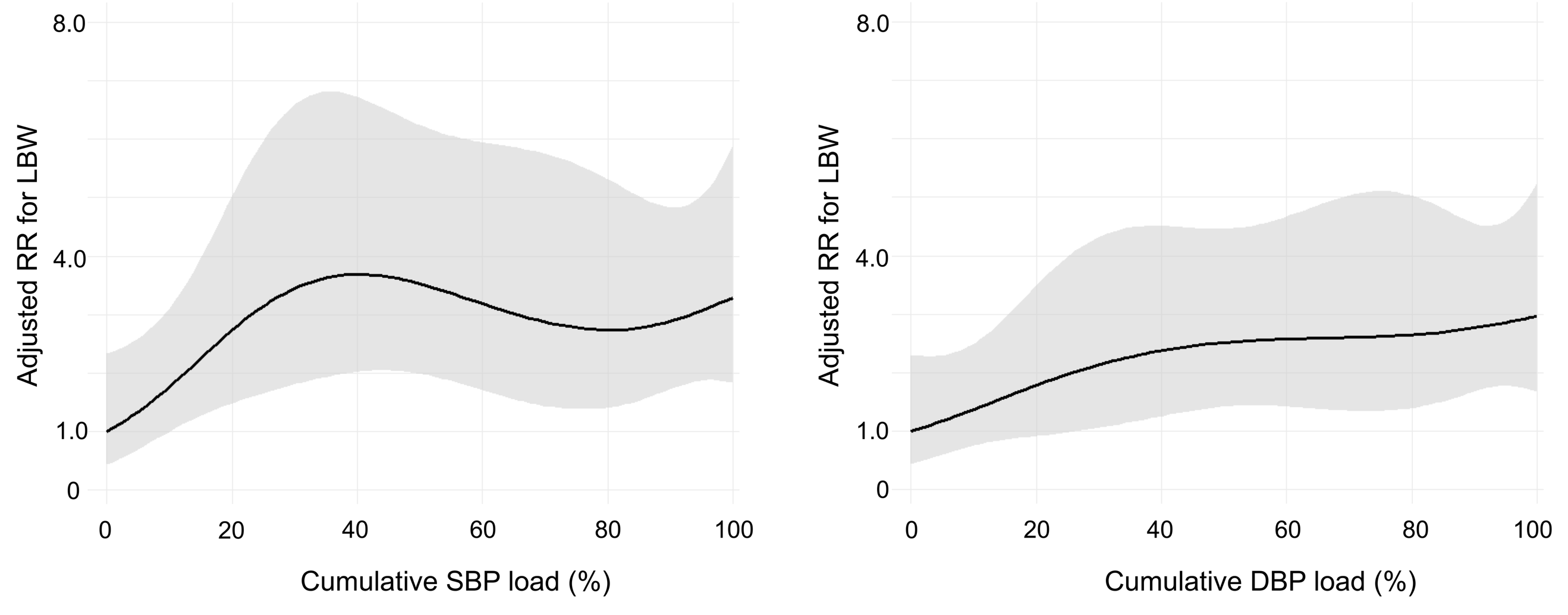


B


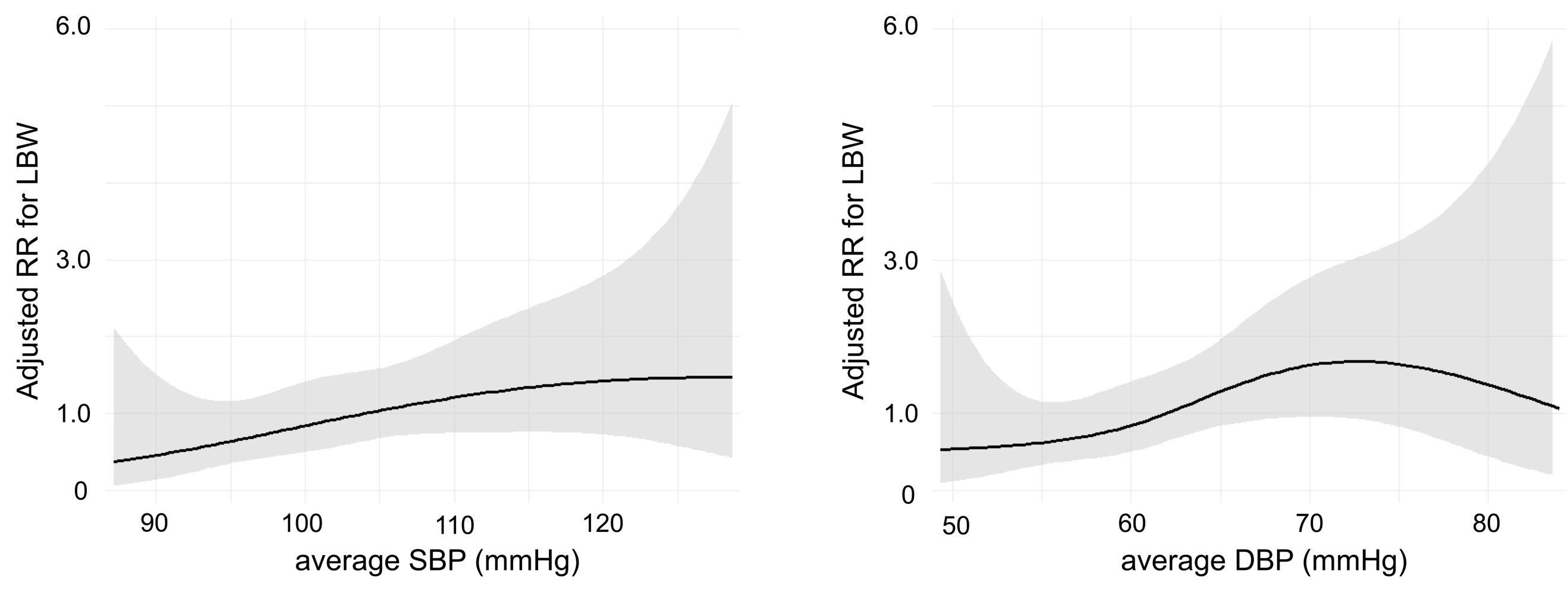


Supplementary Figure 2. Adjusted association with low birth weight using cubic spline modeling. A: Association between the cumulative blood pressure load and the risk of low birth weight. The minimum values of cumulative SBP and DBP load were used as the reference points (relative risk = 1.0). B: Association between the average blood pressure and the risk of low birth weight. The median value of mean systolic blood pressure (104 mmHg) and mean diastolic blood pressure (62 mmHg) was used as the reference (relative risk = 1.0). Relative risks were adjusted for maternal age at gestation, pre-pregnancy body mass index, pre-pregnancy smoking status, primiparity, and history of hypertensive disorders of pregnancy. Gray-shaded areas indicate the 95% confidence intervals. Abbreviations: DBP, diastolic blood pressure; LBW, low birth weight; RR, relative risk; SBP, systolic blood pressure


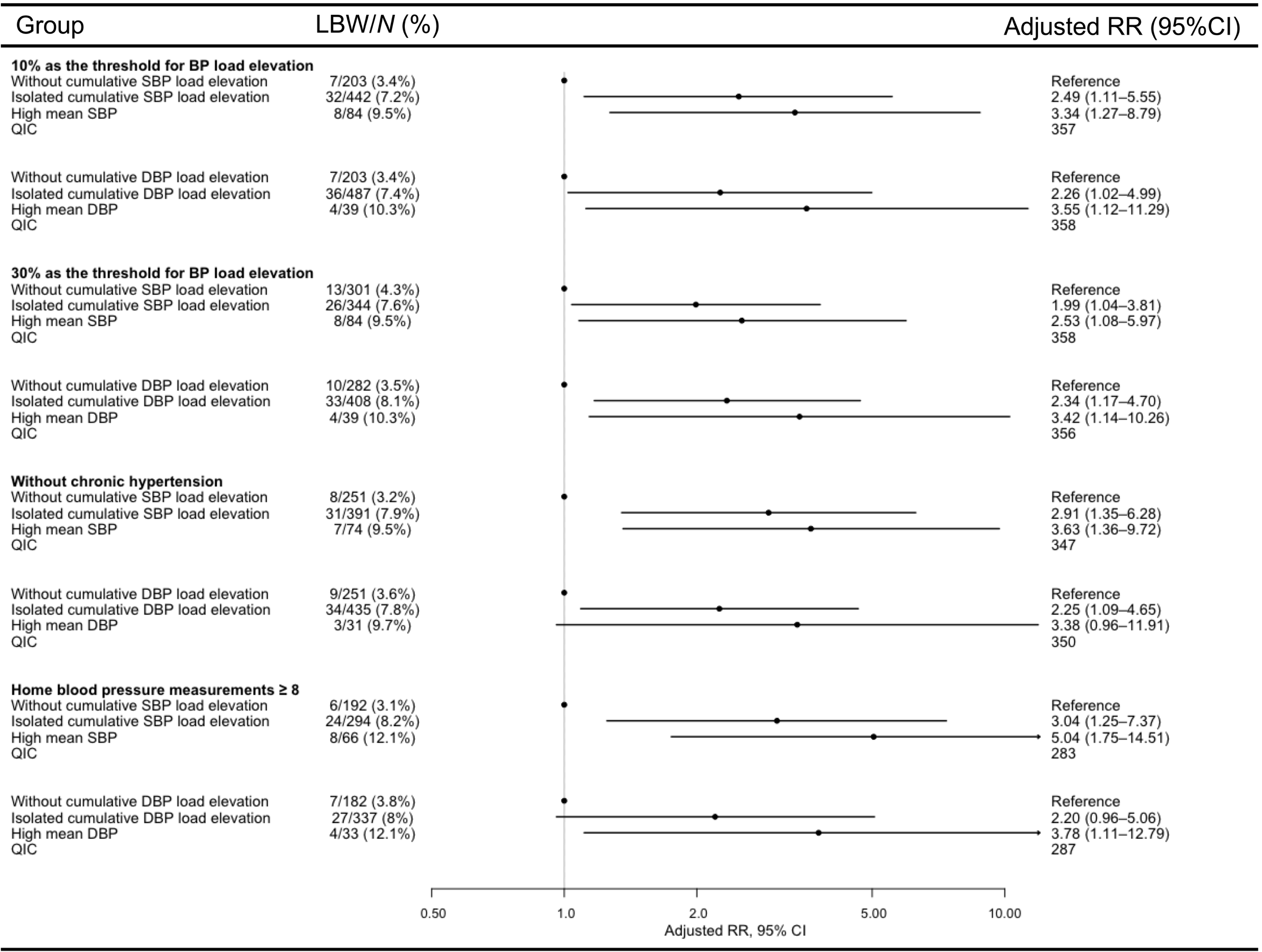


Supplementary Figure 3. Risk assessment of low birth weight based on different cut-offs for cumulative blood pressure load elevation, complication of chronic hypertension, and number of BP measurements. Risk ratios were adjusted for maternal age at gestation, pre-pregnancy body mass index, pre-pregnancy smoking status, primiparity, and history of hypertensive disorders of pregnancy. Abbreviations: BP, blood pressure; CI, confidence interval; DBP, diastolic blood pressure; LBW, low birth weight; QIC, Quasi-likelihood under the Independence model Criterion; RR, risk ratio; SBP, systolic blood pressure
